# A prediction model for major adverse cardiovascular events in patients with heart failure based on high-throughput echocardiographic data

**DOI:** 10.3389/fcvm.2022.1022658

**Published:** 2022-10-28

**Authors:** Qinliang Sun, Shuangquan Jiang, Xudong Wang, Jingchun Zhang, Yi Li, Jiawei Tian, Hairu Li

**Affiliations:** ^1^Department of Ultrasound Imaging, The Second Affiliated Hospital of Harbin Medical University, Harbin, China; ^2^Department of Gastroenterology, Digestive Disease Hospital, Heilongjiang Provincial Hospital Affiliated to Harbin Institute of Technology, Harbin, China

**Keywords:** speckle tracking, vector flow mapping, heart failure, prediction model, nomogram

## Abstract

**Background:**

Heart failure (HF) is a serious end-stage condition of various heart diseases with increasing frequency. Few studies have combined clinical features with high-throughput echocardiographic data to assess the risk of major cardiovascular events (MACE) in patients with heart failure. In this study, we assessed the relationship between these factors and heart failure to develop a practical and accurate prognostic dynamic nomogram model to identify high-risk groups of heart failure and ultimately provide tailored treatment options.

**Materials and methods:**

We conducted a prospective study of 468 patients with heart failure and established a clinical predictive model. Modeling to predict risk of MACE in heart failure patients within 6 months after discharge obtained 320 features including general clinical data, laboratory examination, 2-dimensional and Doppler measurements, left ventricular (LV) and left atrial (LA) speckle tracking echocardiography (STE), and left ventricular vector flow mapping (VFM) data, were obtained by building a model to predict the risk of MACE within 6 months of discharge for patients with heart failure. In addition, the addition of machine learning models also confirmed the necessity of increasing the STE and VFM parameters.

**Results:**

Through regular follow-up 6 months after discharge, MACE occurred in 156 patients (33.3%). The prediction model showed good discrimination C-statistic value, 0.876 (*p* < 0.05), which indicated good identical calibration and clinical efficacy. In multiple datasets, through machine learning multi-model comparison, we found that the area under curve (AUC) of the model with VFM and STE parameters was higher, which was more significant with the XGboost model.

**Conclusion:**

In this study, we developed a prediction model and nomogram to estimate the risk of MACE within 6 months of discharge among patients with heart failure. The results of this study can provide a reference for clinical physicians for detection of the risk of MACE in terms of clinical characteristics, cardiac structure and function, hemodynamics, and enable its prompt management, which is a convenient, practical and effective clinical decision-making tool for providing accurate prognosis.

## Introduction

Urbanization and the widespread use of cars have shifted many people from active to sedentary lifestyles, increasing the incidence of chronic diseases such as obesity, hypertension, diabetes, and coronary artery disease. Heart failure is a serious manifestation of the late stage of various heart diseases, and its risk factors include coronary diseases, hypertension, and diabetes, lifestyle factors such as smoking and alcohol consumption. Heart failure remains a serious clinical and public health problem as the total number of patients living with heart failure increases, reflecting the chronic course of the disease as well as population growth and aging (1). With high readmission and mortality rate, heart failure places a huge financial burden on the healthcare system (2–4).

All efforts must be underway to examine clinical, laboratory, and imaging data to better characterize heart failure phenotypes (5, 6) and to develop cost-effective strategies to reliably identify at-risk populations at an early stage. Therefore, early identification of individuals with high-risk factors will provide an opportunity for the early intervention and prevention of MACE in these individuals. Stratifying patients according to risk of future outcomes and optimizing treatment strategies can help reduce their follow-up costs and mortality (7–9).

The 2019 American College of Cardiology expert consensus on heart failure suggests that assessing risk-increasing factors can help inform decisions about preventive interventions (10). Many risk prediction models have been published internationally (11–16). Predictors of hospitalization rates were age, history of hospitalization for heart failure, edema, systolic blood pressure, and estimated glomerular filtration rate (11). In 2019, the Korean Acute Heart Failure Registry establishes a risk score that predicts the risk of HF specific readmission or death at 30 days after discharge by using 12 predictors (12). In this study, Wang Lei developed a prediction model and nomogram to estimate the risk of irreversible worsening of cardiac function among acute decompensated HF patients, which provided a reference for clinicians to detect and treat cardiac deterioration in a timely manner (13). Compared with traditional HF risk and non-race-specific machine learning models, Race-specific and ML-based HF risk models that combine clinical, laboratory, and biomarker data demonstrated superior performance which can identify distinct race-specific contributors of HF (14). A multivariate Cox regression model has been developed and validated to predict long-term mortality and readmission risk of Chinese patients with chronic heart failure (15). A convenient and accurate prognostic dynamic nomogram model for the risk of all-cause death in acute heart failure patients was developed by Yin T, et al., which included N-terminal pro-brain natriuretic peptide (NT-pro BNP) and growth stimulation expresses gene 2 proteins (16).

However, most of these models are insufficient to reflect the overall situation of the patient. The aim of this study was to develop a predictive model and a predictive nomogram model to estimate the risk of MACE within 6 months of discharge among patients with HF taking into account clinical characteristics, laboratory parameters of blood tests, speckle-tracking echocardiographic analysis, hemodynamic analysis, which can better represent the structure and function of the heart.

## Materials and methods

### Study cohort and study protocol

**FIGURE 1:**
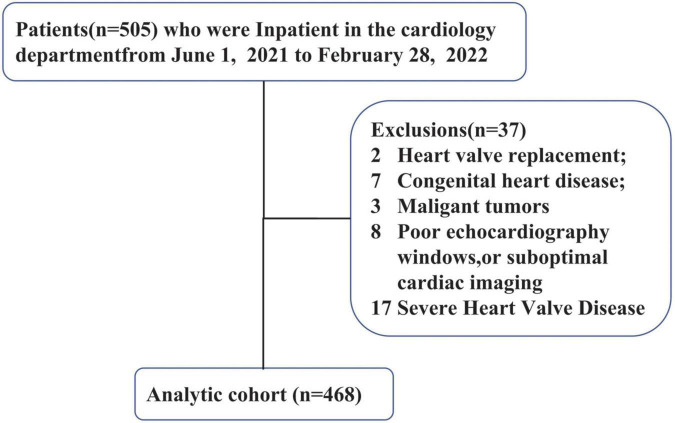
Flowchart demonstrating the process of selection from a total of 504 patients.

This was a single center study. Between July 2021 and February 2022, 505 consecutive patients who were previously diagnosed with chronic heart failure referred to our institution for routine evaluation were screened for eligibility for this study. Criteria and definitions used in the diagnosis of heart failure followed the 2021 heart failure guidelines (17). Inclusion criteria were: (1) consecutive patients who were previously diagnosed with chronic heart failure; (2) pharmacologic therapy followed Guideline Determined Medication Therapy (GDMT) criteria before enrollment in the study. Exclusion criteria were: (1) patients younger than 18 years; (2) history of heart valve replacement, congenital heart disease, severe heart Valve disease, malignant tumors. (3) poor echocardiography windows, or suboptimal cardiac imaging. According to the criteria, 468 patients with HF were finally included (Figure 1). Heart failure patients were classified into the following four categories according to LVEF, including heart failure with preserved ejection fraction (HFpEF), heart failure with mildly reduced ejection fraction (HFmrEF), heart failure with reduced ejection fraction (HFrEF), heart failure with improved ejection fraction (HFimpEF).

All scans were performed by a VFM imaging specialist, and all images were post-processing and analyzed by two professional VFM researchers. Eligible patients were prospectively followed up at 28 days, 3 months, and 6 months after discharge. The study protocol followed the Declaration of Helsinki and was approved by the Research Ethics Committee. All subjects gave written informed consent for additional research tests and for the use of their data for research purposes.

### Standard echocardiography

All echocardiographic examinations follow the guidelines of the American Society of Echocardiography and use commercially available ultrasound equipment (LISENDO 880, Hitachi Healthcare America, Twinsburg, Ohio, USA) (18, 19).

### Speckle-tracking echocardiography

**FIGURE 2:**
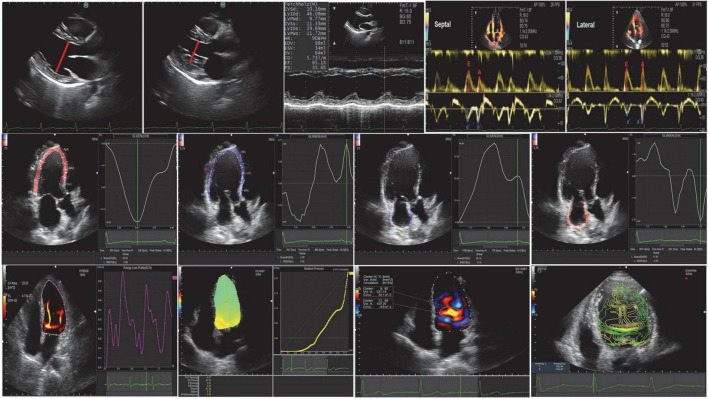
Conventional echocardiography, speckle-tracking echocardiography, and vortex flow mapping echocardiography.

Left atrial (LA) and LV endocardial boundaries were manually determined using QRS early end-diastolic frames as a reference for image analysis (19). The trace can be adjusted manually if necessary. The LV global longitudinal strain (GLS), the LV global systolic strain rate (GLSR) were calculated by the software from 4-, 3-, and 2-chamber images, and averaged for GLS and GLSR. We defined the following components of LA strain: LA reservoir strain = peak (maximal) longitudinal LA strain; LA pump strain = longitudinal LA strain measured between onset of the P wave and onset of the QRS complex; and LA conduit strain = LA reservoir strain–LA pump strain (20). Peak atrial longitudinal strain (PALS) represented LA reservoir function, and peak atrial contraction strain represented LA pump function, which were measured from the average of the strain curves of all segments at the end of ventricular systole. Strain rate analysis was used to measure the peak LA strain rate during the same time-phase divisions described above. Oana Mirea assess the level of agreement between non-dedicated (left ventricular tracking software) and novel dedicated tracking software for RV and LA strain, and found left atrial mean values showed no statistical difference when obtained with the two tracking tools (21; Figure 2).

### Vector flow mapping

Images were analyzed using commercially available off-line software (DASRS1, Hitachi Aloka Medical Ltd., Tokyo, Japan). Using the initial point of the QRS complex as a reference point for image analysis, the LV endocardial border was tracked manually in end-diastolic frames and automatically by the software. Previous studies have limited validation studies (22–26), but the published data may contain useful clinical features. They included: (1) indexes of vortex from the flow-velocity curve, such as vortex area, circulation, maximum vorticity, and the vortex was automatically tracked and analyzed throughout the cardiac cycle (27, 28); (2) energy loss (EL) and mean energy loss (MEL), which were calculated as peak values (23, 24); (3) the intraventricular pressure difference (IVPD) and the intraventricular pressure gradient (IVPG), which were measured on a line that went through the center of the LV from the base to the apex (28, 29). The STE and VFM analysis were post-processed and analyzed by two professional VFM researchers (QL. SUN, Y. LI) (Figure 2).

### Follow-up and clinical outcome data

Follow-up information was obtained during clinical consultations and patients who did not attend scheduled consultations were contacted through telephone interviews with family members. The primary endpoint of the study was the incidence of major adverse cardiac events (MACE). MACE was defined as a complex of congestive HF hospitalization, non-fatal myocardial infarction (MI), non-fatal stroke, and cardiovascular (CV) death. If there are multiple events, the first event is timed for analysis. Eligible patients were prospectively followed up at 28 days, 3 months, and 6 months after discharge. Clinical and echocardiographic parameters were tested for prediction of MACE in the study population. Clinical data, including clinical symptoms and signs, comorbidities, laboratory test results, treatment during hospitalization, and clinical outcomes were obtained by reviewing each patient’s medical records.

### Statistical analysis

We used R (R Foundation, Vienna, Austria) and Python (Python Software Foundation, Beaverton, Oregon, USA) for statistical analysis. Continuous variables are expressed as mean ± SD, frequencies (percentages) for categorical variables and medians (interquartile ranges, IQRs) for skewed variables.

The *t*-test was used to compare the means of continuous variables when the data were normally distributed, else, the Mann-Whitney *U* test was used. One-way analysis of variance (ANOVA) was used for comparisons among multiple groups. Categorical data comparisons between groups were performed using the χ2 test. The statistical significance level for each test was set at α = 0.05; *P* < 0.05 (two-tailed) was considered statistically significant. The Least Absolute Shrinkage and Selection Operator (LASSO) regression algorithm and 10-fold cross-validation were used to filter out the best variables most associated with 6-month MACE incidence. The importance of variables is sorted by machine learning, a logistic regression model is constructed after screening through Venn diagrams, and generates nomograms. We used the Kaplan-Meier method to compare survival between groups. The concordance index (C-index) was used to measure the discriminative abilities of the nomograms [Harrell et al. (30)]. Calibration was performed by examining the survival probability plot predicted by the nomogram.

## Results

### Study population

A total of 468 patients were included, with an average age of 62 years, including 321 males (47.30%). There were 156 cases of MACE, the incidence rate was 33.3%. Patients are divided into four categories based on LVEF: including HFpEF (147 patients, 31.4%), HFmrEF (95 patients, 20.29%), HFrEF (136 patients, 29.06%), HFimpEF (90 patients, 19.23%). Demographic and baseline criteria are detailed by MACE (Table 1) and by LVEF classification (Table 2). Echocardiographic characteristics of STE parameters and VFM parameters are detailed by MACE (Table 3) and by LVEF classification (Table 4).

**TABLE 1:** Baseline characteristics for heart failure (HF) patients during follow-up divided by major cardiovascular events (MACE).

Variables	Overall (*n* = 468)	No event at follow-up (*n* = 312)	Event at follow-up (*n* = 156)	Statistics	*P* value
Patient characteristics					
Sex, male, n (%)	321 (68.59)	212 (67.95)	109 (69.87)	0.18	0.673
Age (years), median [IQR]	62.00 [53.00, 69.00]	62.00 [53.00, 69.00]	62.00 [53.00, 68.00]	–0.02	0.982
BSA (m^2^), median [IQR]	1.79 [1.68, 1.93]	1.79 [1.68, 1.93]	1.78 [1.66, 1.95]	0.28	0.783
DBP (mmHg), median [IQR]	90.00 [80.00, 100.00]	90.00 [80.00, 101.00]	90.00 [80.00, 100.00]	1.85	0.063
SBP (mmHg), median [IQR]	150.00 [120.00, 170.00]	150.00 [126.00, 170.00]	140.00 [120.00, 170.00]	2.65	0.008
Smoke, n (%)	213 (45.51)	136 (43.59)	77 (49.36)	1.4	0.237
Alcohol, n (%)	134 (28.63)	85 (27.24)	49 (31.41)	0.88	0.347
HR (bpm), median [IQR]	79.00 [70.00, 90.00]	78.00 [69.00, 88.00]	81.00 [71.00, 92.00]	–1.88	0.059
NYHA class, n (%)				63.19	< 0.001
I	145 (30.98)	127 (40.71)	18 (11.54)		
II	96 (20.51)	70 (22.44)	26 (16.67)		
III	114 (24.36)	67 (21.47)	47 (30.13)		
IV	113 (24.15)	48 (15.38)	65 (41.67)		
LVEF classification, n (%)				129.42	< 0.001
HFpEF, n (%)	147 (31.41)	127 (40.71)	20 (12.82)		
HFmrEF, n (%)	95 (20.29)	63 (20.19)	32 (20.51)		
HFrEF, n (%)	136 (29.06)	41 (13.14)	95 (60.90)		
HFimpEF, n (%)	90 (19.23)	81 (25.96)	9 (5.77)		
Medical history					
Hypertension, n (%)	332 (70.94)	231 (74.04)	101 (64.74)	4.36	0.037
Hyperlipidemia, n (%)	233 (49.79)	171 (54.81)	62 (39.74)	9.44	0.002
Diabetes mellitus, n (%)	161 (34.40)	114 (36.54)	47 (30.13)	1.89	0.169
HCM, n (%)	16 (3.42)	15 (4.81)	1 (0.64)	5.47	0.019
DCM, n (%)	68 (14.53)	32 (10.26)	36 (23.08)	13.76	< 0.001
Ischemic cardiomyopathy, n (%)	200 (42.74)	123 (39.42)	77 (49.36)	4.2	0.041
Atrial fibrillation, n (%)	105 (22.44)	60 (19.23)	45 (28.85)	5.53	0.019
Medications					
ACEI/ARBS, n (%)	249 (53.21)	167 (53.53)	82 (52.56)	0.04	0.844
ARNI, n (%)	230 (49.15)	154 (49.36)	76 (48.72)	0.02	0.896
β-blockers, n (%)	440 (94.02)	292 (93.59)	148 (94.87)	0.3	0.581
Aldosterone antagonists, n (%)	431 (92.09)	289 (92.63)	142 (91.03)	0.37	0.545
SGLT-2 inhibitors, n (%)	204 (43.59)	128 (41.03)	76 (48.72)	2.5	0.114
diuretics, n (%)	270 (57.69)	148 (47.44)	122 (78.21)	40.34	< 0.001
Antiplatelets, n (%)	305 (65.17)	190 (60.90)	115 (73.72)	7.53	0.006
Anticoagulants, n (%)	268 (57.26)	173 (55.45)	95 (60.90)	1.26	0.261
Statins, n (%)	144 (30.77)	102 (32.69)	42 (26.92)	1.63	0.202
Laboratory examinations					
Glycatedhemoglobin (%), median [IQR]	6.00 [5.60, 7.20]	6.10 [5.50, 7.10]	6.00 [5.60, 7.50]	0.54	0.587
ApolipoproteinA (g/L), median [IQR]	1.15 [1.00, 1.32]	1.18 [1.05, 1.35]	1.07 [0.92, 1.23]	4.39	< 0.001
ApolipoproteinB (g/L), median [IQR]	0.92 [0.72, 1.10]	0.90 [0.72, 1.10]	0.96 [0.73, 1.11]	–0.7	0.483
Inorganicphosphorus (mmol/L), median [IQR]	1.07 [0.93, 1.19]	1.04 [0.90, 1.17]	1.11 [0.98, 1.22]	–3.29	< 0.001
Na (mmol/L), median [IQR]	139.00 [137.00, 141.40]	139.00 [137.00, 141.20]	138.60 [136.00, 141.90]	1.68	0.092
INR, median [IQR]	1.03 [0.97, 1.12]	1.02 [0.96, 1.09]	1.06 [1.00, 1.20]	–4.51	< 0.001
AST (U/L), median [IQR]	22.00 [17.00, 31.00]	21.00 [16.00, 29.00]	25.00 [19.00, 35.00]	–3.48	< 0.001
ALT (U/L), median [IQR]	24.00 [16.00, 35.00]	24.00 [17.00, 35.00]	23.00 [15.00, 36.00]	0.71	0.478
Creatin (umol/L), median [IQR]	84.00 [69.00, 102.00]	82.00 [69.00, 98.00]	87.00 [72.00, 116.00]	–2.58	0.01
Urea/Crea, median [IQR]	83.96 [71.56, 100.66]	83.14 [70.79, 100.66]	86.91 [73.03, 100.61]	–0.73	0.468
Plasma D-dimer (ng/mL), median [IQR]	125.00 [67.00, 263.00]	119.00 [61.00, 235.00]	155.00 [84.00, 323.00]	–3.08	0.002
Fibrinogenr (g/L), median [IQR]	3.01 [2.59, 3.49]	2.98 [2.59, 3.41]	3.11 [2.62, 3.63]	–1.67	0.095
SerumcalciumproteinI (ug/L), median [IQR]	0.11 [0.08, 0.14]	0.11 [0.08, 0.14]	0.11 [0.08, 0.13]	–0.33	0.74
NTproBNP (pg/mL), median [IQR]	971.00 [303.0, 2383.00]	626.00 [223.0, 1565.00]	2372.00 [902.0, 6245.0]	–8.77	< 0.001
General echocardiographic data					
LVESd (mm), median [IQR]	44.40 [31.30, 53.30]	39.70 [29.10, 50.00]	51.20 [42.10, 59.20]	–7.59	< 0.001
LVEDd (mm), median [IQR]	55.80 [48.30, 64.20]	53.10 [47.30, 60.80]	61.80 [54.40, 69.40]	–7.05	< 0.001
LAD (mm), median [IQR]	40.50 [37.00, 44.30]	39.80 [36.30, 42.80]	42.70 [39.00, 46.60]	–5.18	< 0.001
LAVI (ml/m2), median [IQR]	35.68 [27.89, 47.82]	33.36 [26.22, 44.71]	40.42 [33.39, 51.36]	–4.62	< 0.001
LVMI (g/m2), median [IQR]	134.25 [107.54, 164.68]	129.41 [99.62, 159.00]	142.01 [122.18, 174.90]	–4.45	< 0.001
E (m/s), median [IQR]	0.80 [0.62, 1.01]	0.78 [0.60, 1.00]	0.88 [0.67, 1.11]	–3.09	0.002
A (m/s), median [IQR]	0.78 [0.33, 0.98]	0.81 [0.51, 1.00]	0.55 [0.00, 0.90]	3.83	< 0.001
E/A, mean (± SD)	1.13 ± 0.81	1.00 ± 0.63	1.42 ± 1.05	–3.89	< 0.001
e’-S (m/s), median [IQR]	4.90 [3.70, 6.20]	5.10 [4.00, 6.40]	4.30 [3.20, 5.70]	4.42	< 0.001
a’-S (m/s), mean (± SD)	7.48 ± 2.54	7.90 ± 2.42	6.53 ± 2.56	4.92	< 0.001
e’-L (m/s), median [IQR]	6.80 [4.90, 9.20]	7.30 [5.20, 9.40]	6.20 [4.20, 8.60]	3.39	< 0.001
a’-L (m/s), mean (± SD)	9.53 ± 3.29	9.88 ± 3.08	8.73 ± 3.58	2.96	0.003
e’/a’-S, mean (± SD)	0.70 ± 0.29	0.69 ± 0.28	0.73 ± 0.32	–1.31	0.191
e’/a’-L, mean (± SD)	0.80 ± 0.44	0.79 ± 0.42	0.84 ± 0.50	–1.02	0.31
TRV (m/s), mean (± SD)	2.90 ± 0.60	2.85 ± 0.59	2.96 ± 0.61	–1.41	0.159
TRPD (mmHg), mean (± SD)	34.96 ± 14.64	33.73 ± 14.50	36.43 ± 14.66	–1.34	0.181
tei-RV, median [IQR]	0.37 [0.29, 0.45]	0.35 [0.27, 0.44]	0.40 [0.33, 0.47]	–4.25	< 0.001
tei-LV, median [IQR]	0.38 [0.31, 0.44]	0.36 [0.29, 0.43]	0.41 [0.37, 0.47]	–5.96	< 0.001
E/e’-RVav, median [IQR]	6.84 [5.39, 8.40]	6.55 [5.16, 8.09]	7.38 [5.85, 9.22]	–4.28	< 0.001
E/e’-LVav, median [IQR]	14.16 [10.50, 18.29]	13.16 [9.66, 16.49]	16.13 [13.20, 21.76]	–6.29	< 0.001
LVEF, median [IQR]	41.80 [32.40, 61.00]	50.20 [36.00, 62.00]	34.70 [27.50, 43.70]	8.34	< 0.001

*p* < 0.05 indicates statistical significance. BSA, body surface area; DBP, diastolic blood pressure; SBP, systolic blood pressure; HR, heart rate; NYHA, New York Heart Association; DCM, Dilated cardiomyopathy; HCM, hypertrophic cardiomyopathy; ACEI, Angiotensin-converting enzyme inhibitors; ARBS, angiotensin receptor blockers; SGLT-2, sodium-dependent glucose transporters 2; INR, international normalized ratio; ALT, alanine transaminase; AST, aspartate transaminase; LVEDd, left ventricular end diastolic diameter; LVESd, left ventricular end systolic diameter; LAD, left atrium diameter; LAVI, left atrial volume index; LVMI, left ventricular mass index; A, late diastolic transmitral flow velocity; E, early diastolic transmitral flow velocity; e’, early diastolic relaxation velocity at septal mitral annular position; L, lateral side; S, septal side; TRV, tricuspid regurgitant flow velocity; TRPD, tricuspid regurgitation pressure difference; LVEF, left ventricular ejection fraction; NA, not available.

**TABLE 2:** Baseline characteristics for heart failure (HF) patients divided by left ventricular end fraction (LVEF) classification.

Variables	Overall (*n* = 468)	HFpEF (*n* = 147)	HFmrEF (*n* = 95)	HFrEF (*n* = 136)	HFimpEF (*n* = 90)	Statistics	*P* value
Patient characteristics							
Sex, male, n (%)	321 (68.59)	96 (65.31)	57 (60.00)	102 (75.00)	66 (73.33)	7.52	0.057
Age (years), median [IQR]	62.00 [53.00, 69.00]	61.00 [53.00, 68.00]	64.00 [55.00, 71.00]	63.00 [53.00, 69.00]	58.00 [52.00, 67.00]	5.59	0.133
BSA (m^2^), median [IQR]	1.79 [1.68, 1.93]	1.80 [1.68, 1.95]	1.79 [1.69, 1.92]	1.78 [1.66, 1.92]	1.80 [1.66, 1.93]	0.94	0.816
DBP (mmHg), median [IQR]	90.00 [80.00, 100.00]	98.00 [90.00, 110.00]	88.00 [78.00, 100.00]	85.00 [75.00, 93.00]	90.00 [80.00, 100.00]	34.84	< 0.001
SBP (mmHg), median [IQR]	150.00 [120.00, 170.00]	160.00 [140.00, 180.00]	146.00 [121.00, 166.00]	130.00 [115.00, 150.00]	150.00 [120.00, 170.00]	54.36	< 0.001
Smoke, n (%)	213 (45.51)	63 (42.86)	46 (48.42)	65 (47.79)	39 (43.33)	1.2	0.753
Alcohol, n (%)	134 (28.63)	36 (24.49)	25 (26.32)	49 (36.03)	24 (26.67)	5.30	0.151
HR (bpm), median [IQR]	79.00 [70.00, 90.00]	77.00 [67.00, 85.00]	77.00 [70.00, 86.00]	85.00 [75.00, 96.00]	78.00 [69.00, 89.00]	26.40	< 0.001
NYHA class, n (%)						NA	NA
I	145 (30.98)	109 (74.15)	10 (10.52)	0 (0.00)	26 (28.89)		
II	96 (20.51)	25 (17.01)	51 (53.68)	10 (7.35)	10 (11.11)		
III	114 (24.36)	12 (8.16)	22 (23.15)	49 (36.03)	31 (34.44)		
IV	113 (24.15)	1 (0.68)	12 (12.63)	77 (56.62)	23 (25.56)		
Medical history							
Hypertension, n (%)	332 (70.94)	125 (85.03)	67 (70.53)	77 (56.62)	63 (70.00)	27.74	< 0.001
Hyperlipidemia, n (%)	233 (49.79)	95 (64.63)	47 (49.47)	47 (34.56)	44 (48.89)	25.60	< 0.001
Diabetes mellitus, n (%)	161 (34.40)	52 (35.37)	37 (38.95)	36 (26.47)	36 (40.00)	5.97	0.113
HCM, n (%)	16 (3.42)	16 (10.88)	0 (0.00)	0 (0.00)	0 (0.00)	NA	NA
DCM, n (%)	68 (14.53)	0 (0.00)	5 (5.26)	51 (37.50)	12 (13.33)	NA	NA
Ischemic cardiomyopathy, n (%)	200 (42.74)	25 (17.01)	60 (63.16)	63 (46.32)	52 (57.78)	64.99	< 0.001
Atrial fibrillation, n (%)	105 (22.44)	23 (15.65)	14 (14.74)	48 (35.29)	20 (22.22)	20.05	< 0.001
Medications							
ACEI/ARBS, n (%)	249 (53.21)	12 7 (86.40)	36 (37.90)	52 (38.24)	34 (37.78)	94.83	< 0.001
ARNI, n (%)	230 (49.15)	26 (17.69)	61 (64.21)	81 (59.56)	62 (68.89)	86.77	< 0.001
β-blockers, n (%)	440 (94.02)	130 (88.44)	92 (96.84)	133 (97.79)	85 (94.44)	12.97	0.005
Aldosterone antagonists, n (%)	431 (92.09)	136 (92.51)	85 (89.47)	126 (92.64)	84 (93.33)	1.18	0.76
SGLT-2 inhibitors, n (%)	204 (43.59)	47 (31.97)	36 (37.90)	78 (57.35)	43 (47.78)	20.44	< 0.001
diuretics, n (%)	270 (57.69)	30 (20.41)	72 (75.79)	116 (85.29)	52 (57.78)	138.92	< 0.001
Antiplatelets, n (%)	305 (65.17)	77 (52.38)	72 (75.79)	92 (67.65)	64 (71.11)	17.08	< 0.001
Anticoagulants, n (%)	268 (57.27)	72 (48.98)	59 (62.11)	85 (62.50)	52 (57.78)	6.57	0.087
Statins, n (%)	144 (30.77)	59 (40.14)	29 (30.53)	30 (22.06)	26 (28.89)	11.05	0.011
Laboratory examinations							
Glycatedhemoglobin (%), median [IQR]	6.00 [5.60, 7.20]	6.00 [5.50, 6.80]	6.10 [5.60, 8.00]	5.90 [5.60, 7.00]	6.00 [5.80, 7.80]	12.61	0.006
ApolipoproteinA (g/L), median [IQR]	1.15 [1.00, 1.32]	1.20 [1.09, 1.38]	1.12 [0.99, 1.35]	1.07 [0.92, 1.21]	1.17 [1.03, 1.31]	27.39	< 0.001
ApolipoproteinB (g/L), median [IQR]	0.92 [0.72, 1.10]	0.94 [0.72, 1.15]	0.90 [0.71, 1.12]	0.93 [0.73, 1.09]	0.87 [0.69, 1.02]	4.71	0.195
Inorganicphosphorus (mmol/L), median [IQR]	1.07 [0.93, 1.19]	1.02 [0.89, 1.15]	1.08 [0.98, 1.18]	1.13 [0.98, 1.22]	1.04 [0.89, 1.19]	16.50	< 0.001
Na (mmol/L), median [IQR]	139.00 [137.00, 141.40]	139.00 [137.00, 142.00]	138.60 [137.00, 141.40]	138.50 [136.30, 141.40]	139.00 [137.30, 141.00]	4.23	0.238
INR, median [IQR]	1.03 [0.97, 1.12]	1.00 [0.96, 1.06]	1.02 [0.97, 1.09]	1.09 [1.02, 1.22]	1.02 [0.96, 1.08]	46.31	< 0.001
AST (U/L), median [IQR]	22.00 [17.00, 31.00]	19.00 [16.00, 26.00]	23.00 [16.00, 34.00]	27.00 [20.00, 36.00]	21.00 [17.00, 29.00]	26.04	< 0.001
ALT (U/L), median [IQR]	24.00 [16.00, 35.00]	21.00 [15.00, 30.00]	26.00 [17.00, 38.00]	24.00 [15.00, 35.00]	25.00 [19.00, 37.00]	6.14	0.11
Creatin (umol/L), median [IQR]	84.00 [69.00, 102.00]	79.00 [66.00, 93.00]	84.00 [68.00, 105.00]	91.00 [75.00, 116.00]	81.00 [71.00, 95.00]	17.69	< 0.001
Urea/Crea, median [IQR]	83.96 [71.56, 100.66]	83.26 [71.45, 105.80]	85.19 [72.39, 99.56]	84.17 [73.03, 99.12]	83.13 [69.46, 102.71]	0.6	0.89
Plasma D-dimer (ng/mL), median [IQR]	125.00 [67.00, 263.00]	87.00 [56.00, 158.00]	142.00 [82.00, 263.00]	186.00 [93.00, 344.00]	110.00 [58.00, 242.00]	33.00	< 0.001
Fibrinogenr (g/L), median [IQR]	3.01 [2.59, 3.49]	2.99 [2.59, 3.38]	3.03 [2.57, 3.58]	3.02 [2.50, 3.63]	2.99 [2.67, 3.49]	0.49	0.92
SerumcalciumproteinI (ug/L), median [IQR]	0.11 [0.08, 0.14]	0.11 [0.08, 0.14]	0.10 [0.08, 0.14]	0.11 [0.08, 0.14]	0.10 [0.07, 0.14]	3.36	0.339
NTproBNP (pg/mL), median [IQR]	971.00 [303.00, 2383.00]	283.00 [52.00, 799.00]	833.00 [452.00, 1980.000]	2959.00 [1551.00, 7144.00]	1030.00 [304.00, 1788.00]	180.37	< 0.001
General echocardiographic data							
LVESd (mm), median [IQR]	44.40 [31.30, 53.30]	28.60 [24.70, 32.30]	43.10 [40.00, 46.30]	56.20 [51.90, 61.10]	47.90 [33.70, 55.00]	303.31	< 0.001
LVEDd (mm), median [IQR]	55.80 [48.30, 64.20]	47.30 [44.20, 49.70]	55.80 [52.800, 6.10]	65.80 [60.60, 71.70]	58.70 [50.50, 65.20]	245.00	< 0.001
LAD (mm), median [IQR]	40.50 [37.00, 44.30]	37.70 [35.00, 41.30]	40.00 [37.30, 42.00]	44.20 [40.30, 48.70]	41.00 [36.50, 44.00]	75.81	< 0.001
LAVI (g/m^2^), median [IQR]	35.69 [27.89, 47.82]	30.65 [25.11, 41.12]	34.20 [26.88, 44.32]	42.07 [34.80, 56.68]	35.15 [27.32, 49.87]	50.32	< 0.001
LVMI (g/m^2^), median [IQR]	134.25 [107.54, 164.68]	108.33 [92.91, 136.72]	127.38 [108.26, 153.64]	158.73 [134.46, 187.16]	138.60 [118.58, 166.09]	96.57	< 0.001
E (m/s), median [IQR]	0.80 [0.62, 1.01]	0.75 [0.60, 0.90]	0.70 [0.60, 0.83]	1.00 [0.80, 1.24]	0.80 [0.61, 1.00]	48.36	< 0.001
A (m/s), median [IQR]	0.78 [0.33, 0.98]	0.84 [0.64, 1.00]	0.84 [0.62, 1.00]	0.42 [0.00, 0.80]	0.78 [0.30, 1.00]	47.41	< 0.001
E/A ratio, mean (± SD)	0.79 [0.64, 1.32]	0.77 [0.64, 0.90]	0.75 [0.60, 0.90]	1.44 [0.73, 2.52]	0.80 [0.65, 1.29]	25.67	< 0.001
e’-S (m/s), median [IQR]	4.90 [3.70, 6.20]	5.60 [4.50, 6.70]	4.50 [3.60, 5.90]	4.50 [3.10, 5.50]	4.80 [3.50, 6.00]	40.54	< 0.001
a’-S (m/s), mean (± SD)	7.50 [5.70, 9.10]	8.7 [7.40, 10.20]	7.00 [5.90, 8.50]	5.50 [4.10, 7.60]	7.40 [5.30, 9.00]	70.43	< 0.001
e’-L (m/s), median [IQR]	6.80 [4.90, 9.20]	8.7 [6.10, 10.30]	6.20 [4.30, 8.40]	6.30 [4.50, 8.70]	6.50 [4.60, 8.80]	35.78	< 0.001
a’-L (m/s), mean (± SD)	9.4 [7.50, 11.60]	10.2 [9.00, 12.10]	9.0 [7.60, 11.40]	8.0 [5.50, 11.10]	8.7 [7.20, 11.10]	28.40	< 0.001
e’/a’-S, mean (± SD)	0.66 [0.52, 0.81]	0.62 [0.52, 0.73]	0.65 [0.46, 0.83]	0.71 [0.59, 0.88]	0.66 [0.46, 0.83]	11.60	0.009
e’/a’-L, mean (± SD)	0.73 [0.52, 0.90]	0.76 [0.59, 0.93]	0.65 [0.47, 0.85]	0.77 [0.54, 0.95]	0.66 [0.49, 0.86]	9.49	0.023
TRV (m/s), mean (± SD)	2.80 [2.40, 3.22]	2.82 [2.42, 3.10]	2.70 [2.400, 2.90]	2.81 [2.52, 3.31]	2.85 [2.40, 3.40]	2.96	0.397
TRP (mmHg), mean (± SD)	31.00 [24.00, 42.00]	31.00 [24.00, 38.00]	29.00 [23.00, 33.00]	32.00 [25.00, 44.00]	32.00 [24.00, 46.00]	4.02	0.26
tei-RV, median [IQR]	0.37 [0.29, 0.45]	0.30 [0.25, 0.38]	0.37 [0.31, 0.43]	0.42 [0.37, 0.49]	0.38 [0.29, 0.44]	77.33	< 0.001
tei-LV, median [IQR]	0.38 [0.31, 0.44]	0.31 [0.26, 0.38]	0.38 [0.34, 0.43]	0.44 [0.39, 0.50]	0.37 [0.32, 0.47]	114.76	< 0.001
E/e’-RVav, median [IQR]	6.84 [5.39, 8.40]	6.19 [5.12, 7.57]	6.71 [5.16, 7.73]	7.43 [5.82, 9.45]	6.98 [5.59, 8.37]	21.51	< 0.001
E/e’-LVav, median [IQR]	14.16 [10.51, 18.29]	10.08 [8.30, 13.62]	14.82 [12.25, 17.09]	17.61 [13.46, 23.88]	15.31 [11.57, 20.62]	113.05	< 0.001
LVEF, median [IQR]	41.80 [32.40, 61.00]	62.00 [61.00, 64.00]	43.40 [41.30, 46.60]	29.30 [26.60, 33.50]	35.50 [30.80, 57.90]	350.79	< 0.001

*p* < 0.05 indicates statistical significance. BSA, body surface area; DBP, diastolic blood pressure; SBP, systolic blood pressure; HR, heart rate; NYHA, New York Heart Association; DCM, Dilated cardiomyopathy; HCM, hypertrophic cardiomyopathy; ACEI, angiotensin-converting enzyme inhibitors; ARBS, angiotensin receptor blockers; SGLT-2, sodium-dependent glucose transporters 2; INR, international normalized ratio; ALT, alanine transaminase; AST, aspartate transaminase; LVEDd, left ventricular end diastolic diameter; LVESd, left ventricular end systolic diameter; LAD, left atrium diameter; LAVI, left atrial volume index; LVMI, left ventricular mass index; A, late diastolic transmitral flow velocity; E, early diastolic transmitral flow velocity; e’, early diastolic relaxation velocity at septal mitral annular position; L, lateral side; S, septal side; TRV, tricuspid regurgitant flow velocity; TRPD, tricuspid regurgitation pressure difference; LVEF, left ventricular ejection fraction; NA, not available.

**TABLE 3:** Echocardiographic characteristics of speckle tracking echocardiography (STE) parameters and vector flow mapping (VFM) parameters divided by major cardiovascular events (MACE).

Variables	Overall (*n* = 468)	No event at follow-up (*n* = 312)	Event at follow-up (*n* = 156)	Statistics	*P* value
STE parameters					
GLS-LV (%), median [IQR]	−10.81 [−14.90, −8.22]	−12.04 [−16.30, −9.47]	−8.64 [−11.28, −6.47]	−8.05	< 0.001
GLSR-sLV (s^–1^), median [IQR]	−0.60 [−0.76, −0.46]	−0.64 [−0.83, −0.49]	−0.50 [−0.62, −0.36]	−6.76	< 0.001
GLSR-edLV (s^–1^), median [IQR]	0.56 [0.40, 0.76]	0.61 [0.44, 0.81]	0.49 [0.35, 0.64]	4.54	< 0.001
GLSR-acLV (s^–1^), median [IQR]	0.49 [0.32, 0.68]	0.51 [0.38, 0.71]	0.42 [0.27, 0.58]	4.13	< 0.001
PALS-reservoir LA (%),median [IQR]	21.14 [12.96, 30.29]	24.08 [15.69, 32.48]	17.11 [10.36, 23.34]	6.16	< 0.001
PALS-conduit LA (%),median [IQR]	9.53 [6.58, 13.45]	10.66 [7.47, 14.37]	7.60 [5.50, 10.30]	6.5	< 0.001
PALS-pump LA (%),median [IQR]	11.42 [4.98, 17.06]	13.39 [5.89, 18.02]	8.81 [4.00, 14.04]	4.65	< 0.001
GLSR-reservoir LA (s^–1^), median [IQR]	0.95 [0.66, 1.37]	1.09 [0.73, 1.46]	0.83 [0.62, 1.08]	5.44	< 0.001
GLSR-conduit LA (s^–1^), median [IQR]	−0.93 [−1.30, −0.69]	−0.99 [−1.38, −0.73]	−0.81 [−1.06, −0.63]	−4.6	< 0.001
GLSR-pump LA (s^–1^), median [IQR]	−1.21 [−1.83, −0.68]	−1.33 [−1.96, −0.78]	−0.99 [−1.61, −0.58]	−4.01	< 0.001
VFM parameters					
MeanELP1 [J/(m^3 s)], median [IQR]	3.05 [1.84, 4.89]	3.16 [2.01, 4.94]	2.90 [1.40, 4.84]	1.91	0.057
MeanELP2 [J/(m^3 s)], median [IQR]	2.35 [1.47, 3.63]	2.49 [1.59, 3.78]	1.97 [1.20, 3.29]	3.22	0.001
MeanELP3 [J/(m^3 s)], median [IQR]	2.46 [1.42, 4.08]	2.54 [1.40, 4.31]	2.41 [1.44, 3.90]	0.86	0.391
MeanELP4 [J/(m^3 s)], median [IQR]	4.36 [2.54, 7.49]	4.45 [2.61, 7.61]	4.18 [2.40, 7.17]	1.48	0.138
MeanELP5 [J/(m^3 s)], median [IQR]	4.40 [2.56, 7.50]	4.79 [2.79, 7.92]	4.04 [2.04, 6.61]	2.69	0.007
EnergyLossP1 [J/(m s e^3)], median [IQR]	20.44 [13.13, 33.81]	20.31 [13.13, 32.68]	20.54 [13.19, 37.08]	0.16	0.872
EnergyLossP2 [J/(m s e^3)], median [IQR]	14.75 [10.38, 23.13]	14.99 [10.38, 23.80]	14.37 [10.69, 22.32]	1.06	0.289
EnergyLossP3 [J/(m s e^3)], median [IQR]	14.69 [8.76, 24.14]	14.15 [7.67, 23.70]	16.15 [9.87, 25.07]	−1.82	0.068
EnergyLossP4 [J/(m s e^3)], median [IQR]	27.11 [16.70, 46.62]	26.46 [16.03, 45.89]	28.40 [18.08, 48.99]	−0.66	0.509
EnergyLossP5 [J/(m s e^3)], median [IQR]	30.26 [18.18, 50.42]	30.98 [19.60, 52.08]	30.01 [16.48, 46.00]	1.01	0.315
IVPDP1 (mmHg), median [IQR]	0.78 [0.47, 1.22]	0.86 [0.52, 1.26]	0.67 [0.40, 1.00]	3.72	< 0.001
IVPDP2 (mmHg), median [IQR]	1.02 [0.71, 1.41]	1.07 [0.71, 1.45]	0.91 [0.71, 1.29]	2.09	0.036
IVPDP3 (mmHg), median [IQR]	−0.36 [−0.60, −0.25]	−0.38 [−0.63, −0.28]	−0.32 [−0.52, −0.20]	−3.28	0.001
IVPDP4 (mmHg), median [IQR]	−0.97 [−1.39, −0.65]	−1.03 [−1.42, −0.70]	−0.86 [−1.17, −0.56]	−3.23	0.001
IVPDP5 (mmHg), median [IQR]	0.52 [0.29, 0.89]	0.53 [0.27, 0.91]	0.51 [0.30, 0.78]	0.46	0.649
IVPGP1 (mmHg/mme^3), median [IQR]	−8.96 [−15.22, −5.53]	−9.91 [−15.97, −6.02]	−7.74 [−12.11, −4.37]	−4.07	< 0.001
IVPGP2 (mmHg/mme^3), median [IQR]	−12.12 [−16.90, −8.30]	−12.84 [−17.95, −8.57]	−10.42 [−15.23, −8.15]	−2.59	0.01
IVPGP3 (mmHg/mme^3), median [IQR]	4.45 [3.00, 7.30]	4.89 [3.37, 7.69]	3.76 [2.47, 6.32]	3.89	< 0.001
IVPGP4 (mmHg/mme^3), median [IQR]	11.98 [7.98, 17.47]	13.25 [8.80, 18.25]	10.15 [6.57, 14.56]	3.97	< 0.001
IVPGP5 (mmHg/mme^3), median [IQR]	−6.46 [−10.95, −3.31]	−6.62 [−11.55, −3.34]	−6.06 [−9.53, −3.30]	−1.02	0.309
VorArea-s (mm^2), median [IQR]	454.22 [354.72, 574.46]	456.49 [344.67, 568.96]	452.18 [367.50, 577.34]	−0.14	0.888
Circulation-s (m^2/s e^3), median [IQR]	16.90 [11.77, 22.03]	17.42 [12.43, 22.27]	16.10 [10.37, 21.37]	1.95	0.051
VorArea-ed (mm^2), median [IQR]	359.00 [257.14, 476.48]	356.72 [248.46, 468.67]	362.95 [291.99, 493.80]	−1.96	0.05
Circulation-ed (m^2/s e^3), median [IQR]	13.13 [7.57, 21.80]	13.03 [7.40, 21.90]	13.90 [7.60, 21.77]	−0.35	0.727
VorArea-ac (mm^2), median [IQR]	428.02 [323.87, 531.24]	425.12 [324.89, 523.87]	441.81 [319.24, 552.93]	−0.41	0.682
Circulation-ac (m^2/s e^3), median [IQR]	18.03 [12.10, 25.53]	18.97 [12.77, 26.57]	15.77 [10.93, 23.57]	2.73	0.006
vorticity-ac-CCW (1/s), median [IQR]	−105.8 [−142.94, −80.24]	−107.2 [−144.99, −84.58]	−103.6 [−140.37, −75.86]	−1.2	0.231
vorticity-ac-CW (1/s), median [IQR]	114.97 [85.83, 149.68]	119.39 [89.86, 153.89]	108.21 [78.40, 145.27]	2	0.045
vorticity-ed-CCW (1/s), median [IQR]	−99.07 [−135.19, −76.44]	−97.37 [−134.90, −74.64]	−101.4 [−136.47, −80.00]	0.58	0.564
vorticity-ed-CW (1/s), median [IQR]	101.54 [72.03, 140.46]	99.37 [71.15, 140.68]	107.40 [76.73, 139.43]	−1.05	0.292
vorticity-s-CCW (1/s), median [IQR]	−100.7 [−130.23, −75.93]	−102.9 [−132.92, −78.65]	−97.6 [−124.15, −68.81]	−1.97	0.049
vorticity-s-CW (1/s), median [IQR]	110.59 [82.79, 149.78]	115.23 [86.19, 152.53]	103.63 [74.75, 144.74]	2.31	0.021

*p* < 0.05 indicates statistical significance. STE, speckle tracking echocardiography; GLS, global longitudinal strain; GLSR, global systolic strain rate; PALS, peak atrial longitudinal strain; VFM, vector flow mapping; MeanEL, mean energy loss; IVPD, intraventricular pressure difference; IVPG, intraventricular pressure gradient; s, systolic; ed, early diastolic; ac, atrium contraction; P1-P5, isovolumic contraction period, ejection period, isovolumetric relaxation period, diastolic filling period, atrium contraction period; CW, clockwise; CCW, counter-clockwise.

**TABLE 4:** Echocardiographic characteristics of speckle tracking echocardiography (STE) parameters and vector flow mapping (VFM) parameters divided by left ventricular end fraction (LVEF) classification.

Variables	Overall (*n* = 468)	HFpEF (*n* = 147)	HFmrEF (*n* = 95)	HFrEF (*n* = 136)	HFimpEF (*n* = 90)	Statistics	*P* value
STE parameters							
GLS-LV (%), median [IQR]	−10.81 [−14.90, −8.22]	−16.25 [−18.74, −13.16]	−11.06 [−12.81, −9.30]	−7.29 [−9.11, −5.99]	−10.07 [−12.64, −8.05]	246.93	< 0.001
GLSR-sLV (s^–1^), median [IQR]	−0.60 [−0.76, −0.46]	−0.83 [−0.96, −0.66]	−0.60 [−0.68, −0.49]	−0.43 [−0.53, −0.35]	−0.55 [−0.70, −0.46]	189.24	< 0.001
GLSR-edLV (s^–1^), median [IQR]	0.56 [0.40, 0.76]	0.76 [0.54, 0.98]	0.55 [0.41, 0.70]	0.46 [0.32, 0.59]	0.49 [0.38, 0.66]	99.02	< 0.001
GLSR-acLV (s^–1^), median [IQR]	0.49 [0.32, 0.68]	0.56 [0.44, 0.76]	0.57 [0.46, 0.72]	0.34 [0.25, 0.49]	0.45 [0.33, 0.61]	77.00	< 0.001
PALS-reservoir LA (%),median [IQR]	21.14 [12.96, 30.29]	30.86 [21.19, 37.80]	23.32 [18.88, 29.11]	12.70 [9.11, 19.07]	18.72 [11.47, 26.94]	151.51	< 0.001
PALS-conduit LA (%), median [IQR]	9.53 [6.58, 13.45]	13.78 [10.92, 18.03]	9.53 [7.25, 12.35]	6.87 [5.01, 9.56]	8.49 [6.48, 10.66]	137.72	< 0.001
PALS-pump LA (%), median [IQR]	11.42 [4.98, 17.06]	16.64 [11.14, 21.79]	13.17 [9.05, 17.06]	5.07 [3.49, 10.55]	9.72 [4.52, 15.41]	103.30	< 0.001
GLSR-reservoir LA (s^–1^), median [IQR]	0.95 [0.66, 1.37]	1.42 [0.96, 1.83]	1.08 [0.82, 1.35]	0.69 [0.55, 0.91]	0.89 [0.62, 1.25]	133.14	< 0.001
GLSR-conduit LA (s^–1^), median [IQR]	−0.93 [−1.30, −0.69]	−1.24 [−1.66, −0.88]	−0.95 [−1.24, −0.73]	−0.72 [−0.96, −0.58]	−0.83 [−1.13, −0.65]	77.08	< 0.001
GLSR-pump LA (s^–1^), median [IQR]	−1.21 [−1.83, −0.68]	−1.60 [−2.28, −1.14]	−1.36 [−1.89, −1.04]	−0.68 [−1.28, −0.48]	−1.00 [−1.61, −0.60]	89.56	< 0.001
VFM parameters							
MeanELP1 [J/(m^3 s)], median [IQR]	3.05 [1.84, 4.89]	3.56 [2.27, 5.68]	3.10 [2.09, 4.24]	2.51 [1.30, 4.30]	2.88 [1.73, 5.22]	18.06	< 0.001
MeanELP2 [J/(m^3 s)], median [IQR]	2.35 [1.47, 3.63]	3.16 [2.14, 4.78]	2.10 [1.52, 2.99]	1.66 [1.08, 3.15]	2.09 [1.32, 3.54]	55.33	< 0.001
MeanELP3 [J/(m^3 s)], median [IQR]	2.46 [1.42, 4.08]	3.16 [1.99, 5.2]	2.13 [1.33, 3.37]	2.29 [1.30, 3.47]	2.15 [1.28, 4.08]	26.55	< 0.001
MeanELP4 [J/(m^3 s)], median [IQR]	4.36 [2.54, 7.49]	4.90 [3.20, 8.04]	4.19 [2.32, 7.10]	3.95 [2.16, 7.28]	4.18 [2.39, 7.47]	10.93	0.012
MeanELP5 [J/(m^3 s)], median [IQR]	4.41 [2.56, 7.50]	5.78 [3.42, 9.42]	4.27 [2.82, 7.01]	3.64 [1.88, 5.77]	3.77 [2.35, 7.23]	34.34	< 0.001
EnergyLossP1 [J/(m s e^3)], median [IQR]	20.45 [13.13, 33.81]	20.20 [13.29, 30.70]	20.86 [14.55, 32.68]	21.61 [12.68, 36.57]	18.96 [12.61, 36.64]	0.40	0.941
EnergyLossP2 [J/(m s e^3)], median [IQR]	14.75 [10.38, 23.13]	16.12 [11.69, 25.93]	14.07 [10.32, 20.20]	15.13 [9.17, 23.80]	13.68 [9.78, 21.71]	6.22	0.102
EnergyLossP3 [J/(m s e^3)], median [IQR]	14.69 [8.76, 24.14]	14.75 [9.58, 24.97]	12.10 [7.88, 19.17]	16.53 [9.54, 25.71]	14.15 [7.52, 23.54]	6.25	0.1
EnergyLossP4 [J/(m s e^3)], median [IQR]	27.11 [16.70, 46.62]	26.06 [15.96, 42.05]	25.75 [15.93, 44.21]	30.35 [20.08, 50.68]	25.60 [17.25, 46.62]	3.67	0.3
EnergyLossP5 [J/(m s e^3)], median [IQR]	30.26 [18.19, 50.42]	31.57 [20.18, 53.73]	29.88 [19.78, 50.33]	28.34 [16.17, 44.75]	26.64 [17.43, 46.89]	3.39	0.336
IVPDP1 (mmHg), median [IQR]	0.78 [0.47, 1.22]	1.13 [0.71, 1.46]	0.71 [0.43, 1.15]	0.62 [0.378, 0.96]	0.71 [0.43, 1.00]	49.71	< 0.001
IVPDP2 (mmHg), median [IQR]	1.02 [0.71, 1.41]	1.12 [0.74, 1.56]	1.05 [0.72, 1.39]	0.90 [0.70, 1.28]	1.020 [0.657, 1.33]	8.35	0.039
IVPDP3 (mmHg), median [IQR]	−0.36 [−0.60, −0.25]	−0.51 [−0.74, −0.33]	−0.31 [−0.43, −0.24]	−0.34 [−0.57, −0.16]	−0.34 [−0.52, −0.24]	37.65	< 0.001
IVPDP4 (mmHg), median [IQR]	−0.97 [−1.39, −0.65]	−1.22 [−1.71, v0.88]	−0.88 [−1.22, −0.63]	−0.81 [−1.16, −0.52]	−0.97 [−1.32, −0.61]	45.79	< 0.001
IVPDP5 (mmHg), median [IQR]	0.52 [0.29, 0.89]	0.67 [0.33, 1.13]	0.61 [0.32, 0.91]	0.45 [0.27, 0.70]	0.45 [0.21, 0.78]	13.80	0.003
IVPGP1 (mmHg/mme^3), median [IQR]	−8.96 [−15.22, −5.53]	−14.52 [−19.48, −8.47]	−8.45 [−13.37, −5.40]	−6.96 [−10.34, −3.93]	−8.35 [−12.47, −5.18]	67.63	< 0.001
IVPGP2 (mmHg/mme^3), median [IQR]	−12.12 [−16.90, −8.30]	−14.45 [−20.48, −9.29]	−12.58 [−16.63, −8.44]	−10.15 [−14.84, −7.49]	−12.21 [−16.32,- −8.01]	22.76	< 0.001
IVPGP3 (mmHg/mme^3), median [IQR]	4.45 [3.00, 7.30]	6.56 [4.38, 10.20]	3.78 [2.96, 5.18]	3.73 [1.99, 6.74]	4.06 [2.95, 6.51]	58.77	< 0.001
IVPGP4 (mmHg/mme^3), median [IQR]	11.98 [7.98, 17.47]	15.80 [11.76, 23.72]	11.01 [7.73, 15.08]	9.23 [5.65, 13.70]	11.46 [7.26, 16.12]	75.75	< 0.001
IVPGP5 (mmHg/mme^3), median [IQR]	−6.46 [−10.95, −3.31]	−9.11 [−14.72, −4.37]	−7.71 [−10.88, −4.05]	−5.04 [−7.80, −2.87]	−5.76 [−9.65, −2.50]	24.53	< 0.001
VorArea-s (mm^2), median [IQR]	454.22 [354.72, 574.46]	423.08 [337.60, 551.43]	460.09 [384.78, 576.38]	461.12 [360.21, 579.91]	465.20 [350.05, 587.72]	3.07	0.381
Circulation-s (m^2/s e^3), median [IQR]	16.90 [11.77, 22.03]	18.57 [14.27, 23.33]	17.13 [12.63, 23.23]	15.03 [8.50, 19.30]	16.10 [10.80, 21.07]	19.85	< 0.001
VorArea-ed (mm^2), median [IQR]	359.00 [257.14, 476.48]	337.17 [225.00, 471.63]	345.53 [263.20, 448.11]	386.44 [284.88, 509.13]	359.00 [289.74, 470.27]	7.96	0.047
Circulation-ed (m^2/s e^3), median [IQR]	13.13 [7.57, 21.80]	14.63 [9.03, 22.30]	12.57 [7.33, 20.87]	12.87 [7.07, 21.17]	13.10 [7.13, 21.73]	2.85	0.416
VorArea-ac (mm^2), median [IQR]	428.02 [323.87, 531.24]	404.47 [312.14, 504.52]	447.10 [338.74, 556.72]	457.42 [346.38, 561.19]	417.28 [324.89, 517.32]	7.85	0.049
Circulation-ac (m^2/s e^3), median [IQR]	18.03 [12.10, 25.53]	19.70 [13.30, 27.10]	19.00 [13.40, 27.13]	15.47 [10.83, 21.93]	17.90 [11.73, 24.73]	10.65	0.014
vorticity-ac-CCW (1/s), median [IQR]	−105.79 [−142.94, −80.24]	−113.56 [−152.41, −89.29]	−103.55 [−148.56, −76.79]	−94.59 [−131.58, −73.93]	−106.5 [−135.9, −86.7]	10.76	0.013
vorticity-ac-CW (1/s), median [IQR]	114.97 [85.83, 149.68]	128.97 [95.50, 165.98]	117.48 [91.04, 151.08]	107.01 [73.69, 133.29]	105.04 [88.66, 137.78]	22.55	< 0.001
vorticity-ed-CCW (1/s), median [IQR]	−99.07 [−135.19, −76.44]	−96.51 [−127.33, −78.97]	−92.34 [−124.11, −69.19]	−107.40 [−151.76, −81.92]	−99.77 [−131.18, −74.86]	7.01	0.072
vorticity-ed-CW (1/s), median [IQR]	101.54 [72.03, 140.46]	98.34 [71.22, 140.46]	98.33 [71.15, 143.29]	115.89 [74.59, 140.68]	95.48 [72.29, 140.17]	2.94	0.401
vorticity-s-CCW (1/s), median [IQR]	−100.69 [−130.23, −75.93]	−112.44 [−145.15, −83.37]	−97.57 [−124.52, −77.99]	−90.01 [−122.77, −67.34]	−104.3 [−134.5, −85.9]	17.34	< 0.001
vorticity-s-CW (1/s), median [IQR]	110.59 [82.79, 149.78]	132.47 [99.22, 173.11]	105.17 [80.06, 147.14]	97.38 [69.16, 131.18]	105.57 [75.87, 144.28]	37.41	< 0.001

*p* < 0.05 indicates statistical significance. STE, speckle tracking echocardiography; GLS, global longitudinal strain; GLSR, global systolic strain rate; PALS, peak atrial longitudinal strain; VFM, vector flow mapping; MeanEL, mean energy loss; IVPD, intraventricular pressure difference; IVPG, intraventricular pressure gradient; s, systolic; ed, early diastolic; ac, atrium contraction; P1-P5, isovolumic contraction period, ejection period, isovolumetric relaxation period, diastolic filling period, atrium contraction period; CW, clockwise; CCW, counter-clockwise.

### Select optimal prognostic variables and model development

**FIGURE 3:**
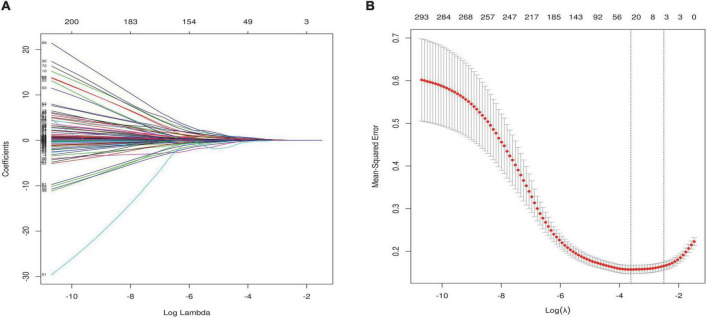
Feature selection using the least absolute shrinkage and selection operator (LASSO) binary logistic regression model. **(A)** LASSO coefficient profiles of the 320 features. Vertical line was drawn at the value selected using 10-fold cross-validation, where optimal resulted in 26 non-zero coefficients. **(B)** Tuning parameter (λ) selection in the LASSO model used 10-fold cross-validation *via* minimum criteria. A coefficient profile plot was produced against the log (λ) sequence. The area under the receiver operating characteristic (AUC) curve was plotted versus log(λ). This is consistent with the glmnet package recommendation for choosing λ, as either λ min (minimum mean square error) or this value plus one standard error.

Feature selection was performed using the LASSO regression algorithm *via* the R package glmnet and 10-fold cross-validation. This is consistent with the glmnet package recommendation for choosing λ, as either λ min (minimum mean square error) or this value plus one standard error. When lambda equaled 0.035, twenty-six optimal prognostic variables were identified, including New York Heart Association (NYHA), hyperlipidemia stability (HPLS), ischemic cardiomyopathy (ICM), age, diastolic blood pressure (DBP), left ventricular end diastolic diameter (LVEDd), left ventricular end systolic diameter (LVESd), peak mitral valve blood flow during atrial contraction (A), LV ejection fraction (LVEF), aortic regurgitation (AR), inorganicphosphorus (P), glycatedhemoglobin (GHB), apolipoproteinB (ApoB), sodium ion (Na), N-terminal brain natriuretic peptide (NTproBNP), international normalized ratio (INR), fibrinogen, cardiac index (CI), LV vortex area from 4-chamber images during systolic period (VorAreaC4S), LV energy loss from 3-chamber images during isovolumic relaxation period (ELC3P3), intraventricular pressure differences from 2-chamber images during LV early filling period (IVPDC2P4), LA global longitudinal strain rate during conduit period (GLSRconduitC4LA), LV global longitudinal strain from 3-chamber images during early diastolic period (GLSedC3LV), LV global longitudinal strain from 2-chamber images during atrial systolic period (GLSacC2LV), average of LV global longitudinal strain during atrial systolic period (GLSacLV), peakLA longitudinal peak strain during conduit period (PALSconduitLA) (Figures 3A,B).

**FIGURE 4:**
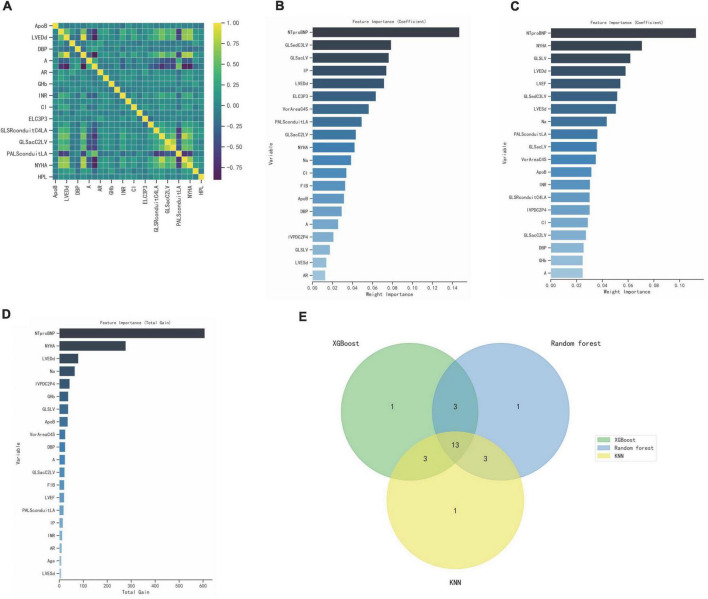
Select optimal prognostic variables. **(A)** Correlation heatmap showing associations between clinical features and ultrasound parameters. **(B–D)** XGBoost, Random forest, KNN calculate the importance of each influencing factor to the classification model and rank it, the top 20 parameters were selected. **(E)** Venn diagram, 13 variables were recognized.

Correlation heatmap showing associations between clinical features and ultrasound parameters after lasso dimension reduction analysis (Figure 4A). Here, we used three different popular machine-learning methods including extreme gradient boosting classifier (XGBoost), random forest classifier, k-nearest neighbor classifier (KNN), which were widely applied in bioinformatics in order to calculate the importance of each influencing factor to the classification model and rank it, the top 20 parameters were selected (Figures 4B–D). R (Venn Diagram package) was employed to generate the Venn diagram, then 13 variables were recognized through the Venn diagram (Figure 4E), including NTproBNP, NYHA, GLSLV, LVEDd, LVESd, A, Na, PALSconduitLA, VorAreaC4S, ApoB, IVPDC2P4, GLSacC2LV, DBP. In this study, LVEF with high clinical acceptance was included, and the variance inflation factor (VIF) was calculated by collinearity analysis. LVESd was excluded because of the higher VIF, and A was excluded because of the higher missing. Finally, 12 candidate predictors were selected to build the prediction model, including NYHA, DBP, NTproBNP, Na,ApoB, LVEF, LVEDd, GLSLV, PALSconduitLA, GLSacC2LV, VorAreaC4S, IVPDC2P4, and built a nomogram based on the logistic regression model (Table 5).

**TABLE 5:** Prognostic model for major cardiovascular events (MACE) based on logistic regression model.

Predictor	Estimate	SE	*Z*	*p*	Odds ratio	Lower (95% CI)	Upper (95% CI)
IVPDC2P4 (mmHg)	0.345	0.163	2.116	0.034	1.411	1.039	1.978
VorAreaC4S (mm^2)	0.001	0.001	1.664	0.046	1.001	1	1.002
GLSacC2LV (%)	0.155	0.079	1.967	0.049	1.168	1.003	1.367
PALSconduitLA (%)	–0.06	0.039	–1.539	0.024	0.942	0.87	1.014
GLSLV (%)	–0.041	0.056	–0.726	0.048	0.96	0.86	1.072
LVEDd (mm)	0.05	0.021	2.379	0.017	1.051	1.009	1.096
LVEF (%)	0.033	0.022	1.495	0.035	1.033	0.99	1.079
ApoB (g/L)	1.145	0.458	2.501	0.012	3.143	1.288	7.821
Na (mmol/L)	–0.056	0.034	–1.674	0.044	0.945	0.885	1.009
NTproBNP (mmol/L)	0.001	0.001	4.745	0.001	1	1	1.001
DBP (mmHg)	–0.014	0.007	–1.972	0.049	0.986	0.971	1
NYHA II	2.263	0.797	2.841	0.005	9.611	2.439	64.577
NYHA III	2.918	0.857	3.406	0.001	18.503	4.052	134.706
NYHA IV	3.367	0.896	3.758	0.001	28.979	5.799	222.979

*p* < 0.05 indicates statistical significance. IVPDC2P4, intraventricular pressure differences from 2-chamber images during LV early filling period; VorAreaC4S, LV vortex area from 4-chamber images during systolic period; GLSacC2LV, left ventricular global longitudinal strain from 2-chamber images during atrial systolic period; PALSconduitLA, peakLA longitudinal peak strain during conduit period; GLSLV, left ventricular global longitudinal strain; LVEDd, left ventricular end diastolic diameter; LVEF, left ventricular ejection fraction; ApoB, apolipoproteinB; NT-pro BNP, N-terminal pro-brain natriuretic peptide; DBP, diastolic blood pressure; NYHA, New York Heart Association.

### Nomogram interpretation and model validation

**FIGURE 5:**
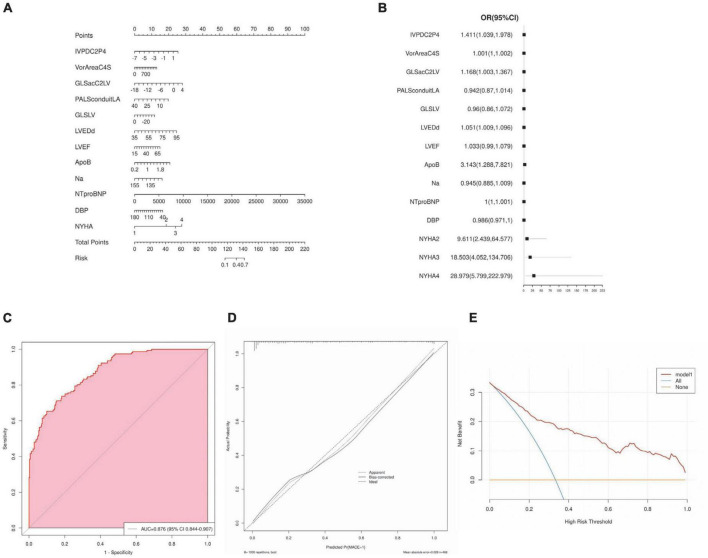
Nomogram interpretation and model validation. **(A)** Nomogram for predicting major cardiovascular events (MACE) risk, and the point was the selected scoring standard or scale. **(B)** Odds ratios determined in a logistic regression model as shown in the forest plot. **(C)** Receiver operating characteristic curve for the nomogram. **(D)** The Nomogram calibration plots of the model. **(E)** Decision curve analysis for predictive models.

Nomogram for predicting MACE risk, and the point was the selected scoring standard or scale. For each independent variable, by drawing a line (through the ruler) perpendicular to the point axis, the intersection points represent the values of the independent variables. The value of each variable is scored on a scale of 0 to 100, and the scores for each variable are summed to estimate the position perpendicular to the axis. That sum enables us to predict the probability of MACE risk in patient with HF (Figure 5A). Estimated odds ratios determined in a logistic regression model as shown in the forest plot (Figure 5B). Receiver operating characteristic curve for the nomogram generated using bootstrap resampling, which showed a good discriminative ability for the prediction model (C-statistics: 0.876 [95% CI, 0.844– 0.907]) (Figure 5C). The Nomogram calibration plots of the model based on the bootstrap method showed good performance (Figure 5D). When the solid line (performance nomogram) was closer to the dotted line (ideal model), the prediction accuracy of the nomogram was better. Decision curve analysis for predictive models (Figure 5E). Solid red line is predictive models, solid blue line is patients with MACE, and solid horizontal line is patients without MACE. The graph shows the expected net benefit per patient in relation to the nomogram MACE risk prediction. Decision curve analysis indicated that the clinical validity of the model was moderate.

### Survival curves based on nomogram scores and predictive risk

**FIGURE 6:**
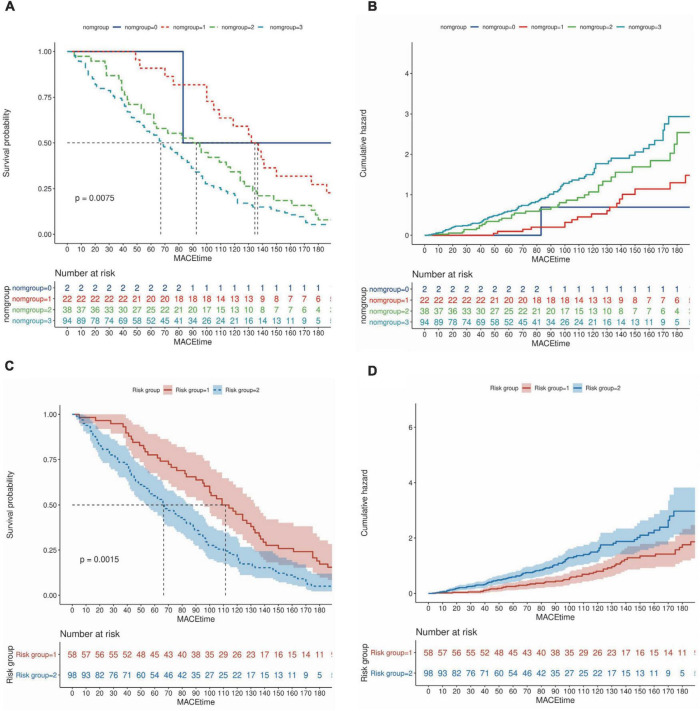
Survival curves based on nomogram scores and predictive risk. **(A,B)** Patients were divided into four groups according to the quartile total nomogram scores (nomgroup = 0, 1, 2, 3), the KM survival curves and cumulative survival curves were used to compare survival times among different groups was made by log-rank test. **(C,D)** Patients were divided into high-‘and low-risk groups (low-risk group = 1, high–risk group = 2) according to the predicted probabilities of the prediction model. The median survival time (LT50) for different groups. The horizontal axis of the KM survival curve represents time, and the vertical axis represents probability.

Patients were divided into four groups according to the quartile total nomogram scores (nomgroup = 0, 1, 2, 3), and each group had 117 patients. A prediction model with a prediction probability of less than 0.5 is considered a low risk group, and patients were divided into high-risk and low-risk groups (low-risk group = 1, high-risk group = 2). Of these, 342 patients (73.08%) belonged to the low-risk group. The KM survival curves and cumulative survival curves were used to compare survival times among different groups was made by log-rank test. The median survival time (LT50) for different groups. The horizontal axis of the KM survival curve represents time, and the vertical axis represents probability (Figure 6).

### Performance of machine learning algorithms among the groups

**FIGURE 7:**
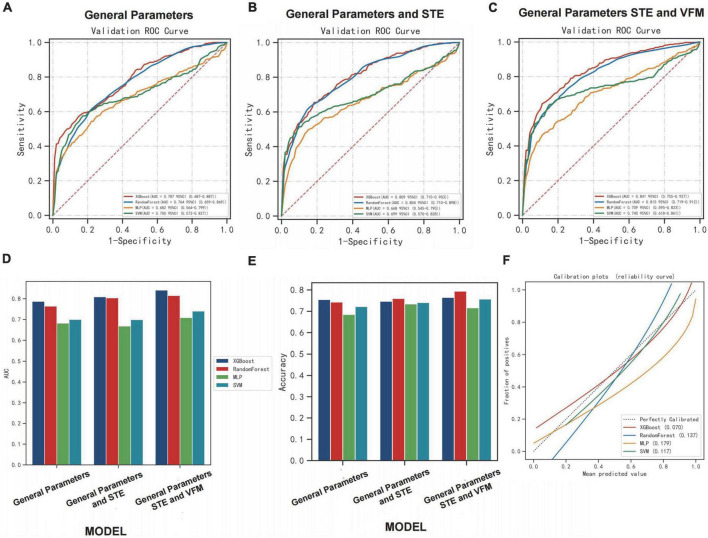
Performance of machine learning algorithms among the groups. **(A–C)** The ROC curve of the model performance in classifying patients correctly in each group using learning algorithms. **(D,E)** The performances [area under curve (AUC) and accuracy] of the model in classifying patients correctly in each group using learning algorithms. **(F)** The calibration plots of learning algorithms in group [General Parameters, speckle tracking echocardiography (STE) and vector flow mapping (VFM) group].

We divided patients into training and validation sets using MACE as the categorical outcome variable. Parameters include general parameters, STE-related parameters and VFM-related parameters. General parameters include basic characterization parameters, clinical parameters, blood laboratory test results, and conventional ultrasound parameters. Four machine learning models were used to complete the data sample classification task, including: XGBoost Classifier, Random Forest Classifier, Multi-LayerPerceptron (MLP) Classifier, support vector machine (SVM) Classifier. By repeated sampling 10 times and each resampling training validation set of 20.0% of the total sample, training set of 80.0%, the model classification was successively performed and drawed the ROC curve. General Parameters group (AUC: 0.79, accuracy: 0.75, AUC: 0.76, accuracy: 0.74, AUC: 0.68, accuracy: 0.69, AUC: 0.70, accuracy: 0.72, respectively), General Parameters and STE group (AUC: 0.81, accuracy: 0.75; AUC: 0.80, accuracy: 0.76, AUC: 0.67, accuracy: 0.73, AUC: 0.70, accuracy: 0.74, respectively), General Parameter, STE and VFM group (AUC: 0.84, accuracy: 0.77, AUC: 0.82, accuracy: 0.79, AUC: 0.71, accuracy: 0.72, AUC: 0.74, accuracy: 0.76, respectively) were classified in the test set performance (Figures 7A–C).

Comparing AUC and accuracy, the XGBoost classifier performed the best among all four learning algorithms. In each group, we found that AUC and accuracy of four learning algorithms were higher when the parameters included general parameters, STE-related parameters and VFM-related parameters, and were more pronounced when using the XGBoost classifier. The inclusion of STE and VFM parameters in the XGBoost classifier model improved its ability to correctly classify MACE patients compared to other learning algorithms (AUC: 0.84, accuracy: 0.77). The inclusion of the STE and VFM parameters in the XGBoost classifiers model showed an improved ability to classify patients with MACE correctly compared with the other learning algorithms, which is more beneficial to predicting the occurrence of MACE. The performances (AUC and accuracy) of the model in classifying patients correctly in each group using learning algorithms are shown (Figures 7D,E). Calibration plot of four learning algorithms (Figure 7F). When the solid line was closer to the dotted line (perfectly calibrated), the prediction accuracy was better. Among the comparison of Brier scores for the above four learning algorithms, XGBoost is the lowest, and his prediction calibration is the best (Brier scores = 0.07).

## Discussion

The primary objective of this study was to develop a scoring system to predict MACE risk in patients with chronic heart failure. This study used clinical trial data from patients with CHF, and age was not included in the risk assessment model due to the short follow-up time. In previous models, the most common parameters used to predict major adverse cardiovascular events were general clinical data, blood test parameters, and conventional ultrasound parameters (11–16). There are also articles that use LA and LV strain parameters to create models. LA function assessed by speckle tracking echocardiography is an independent prognostic marker in heart failure patients with reduced ejection fraction (31). These results suggest that STE variable information corresponding to two-dimensional and doppler analysis can provide independent assessments of diastolic function and LV filling pressures, which are increasingly moving toward precision medicine (32).

Left atrial (LA) function is closely related to LV function and plays a key role in maintaining optimal cardiac functional performance. The left atrium, through its reservoir, conduit, and booster pump stages, regulates filling of the left ventricle, while the LV influences LA function throughout the cardiac cycle. Long-term exposure to high LV filling pressures leads to an increase in LA volume, presumably reduced LA function is only a marker of LV deterioration (33, 34). In patients with HFrEF, LA function index measured at first admission was associated with cardiovascular outcomes during the first 6 months of follow-up (35), and LA systolic function measured by LA strain rate was shown to provide prognostic stratification for outpatients with new-onset HF (36). The prognostic value of LA function has also been explored using other imaging techniques. Decreased LA ejection fraction measured by cardiac magnetic resonance imaging was associated with a higher incidence of AF and poorer prognosis in patients with HF (37). Previous studies have shown that LV diastolic dysfunction is related to LA volume (38, 39). Moreover, LV diastolic function is closely related to atrial function; Thus, the transmitral flow parameters of LV must be interpreted in terms of LV relaxation, LA function, and loading parameters, each of which affects LV filling pressure. Due to the high dimensionality and complexity of variables affecting LV diastolic function and filling pressures, echocardiographic assessment requires a multiparametric approach (19, 40). Heart failure patients are divided into four categories based on LVEF: HFpEF, HFmrEF, HFrEF HFimpEF (17). Different subtypes have different clinical features and disease courses, and different conditions can lead to misjudgment of heart failure and bring difficulties to the diagnosis of heart failure. It is well-known that HFpEF differs in clinical features and disease course (including treatment strategies), but all-cause readmission rates remained the highest in HFpEF vs. HFrEF and HFmrEF (41). This study hopes to establish a predictive model suitable for different heart failure populations, and only LVEF analysis variables are included, not LVEF classification. We will continue to expand the sample size and construct respective prediction models for different types of heart failure patients, so as to improve the prediction effect of the model.

Currently, left chamber fluid mechanics is lacking to participate in model construction. There is a growing interest in the imaging and visualization of intracardiac blood flow (42). VFM is a two-dimensional cross-sectional image acquired based on b-type color Doppler echocardiography, which can enables visualize the cross-sectional image with blood flow as a velocity vector (43). As mentioned earlier, calculate the flow vector using the continuity equation and decompose the vector into vertical and parallel velocity components, determined from wall motion spot tracking and color doppler images (25). The advantages of VFM are that it is relatively inexpensive, less time-consuming, easy to use at the bedside, and does not require the use of contrast agents. This model adds the VFM parameters compared with the previous models, which makes the significance of the model more explanatory and the evaluation more comprehensive. The potential application of VFM to quantify aortic regurgitation has been reported. Compared with other conventional measures, energy loss can more clearly quantify patients, subjective symptoms and help to assess disease severity based on cardiac stress (24, 44, 45). Automated phenotype calculation is an efficient strategy to fuse multidimensional parameters of LV structure and function to collect STE- and CFM-related parameters (46).

In this study, the left ventricular high-throughput parameters were constructed by collecting mechanics and hydrodynamic parameters, including STE parameters of LV and LA, VFM parameters of LV flow. For the first time, we integrate multiple echocardiographic parameters to meaningfully reflect changes in left ventricular structure and function. Through screening, key indicators are incorporated into the construction of the model. Based on selecting optimal prognostic variables, a logistic regression model and nomogram is constructed, which can well-predict the possibility of MACE within 6 months. Here, we evaluated the relationship between these factors and HF to create a practical and accurate prognostic dynamic nomogram model to identify high-risk groups of heart failure and ultimately develop targeted treatment options. From Figure 5C, we can find the performance effect of constructing the new model is good [C-statistics: 0.876 (95% CI, 0.844– 0.907)]. From Figure 6, survival curves were generated using the Kaplan–Meier method, and log-rank tests were used to compare survival curves, which based on nomogram scores and predictive risk. For survival analysis, significant differences among the groups were seen according to Kaplan–Meier survival curves. We can find patients with high nomogram scores and predictive risk had the shortest survival time. Although the number of high-risk group was significantly fewer than low risk group, the number of patients with MACE was significantly more than the low risk group, better demonstrating the clinical significance of the model. In each group, we found that AUC and accuracy of four learning algorithms were higher when the parameters included general parameters, STE-related parameters and VFM-related parameters, and were more pronounced when using the XGBoost classifier. The inclusion of general parameters and STE-related parameters and VFM-related parameters in the XGBoost classifiers model showed an improved ability to classify patients with MACE correctly compared with the other learning algorithms (AUC: 0.84, accuracy: 0.77), which is more beneficial to predicting the occurrence of MACE. Calibration plot of four learning algorithms, we can find among the comparison of Brier scores, XGBoost is the lowest, and his prediction calibration is the best (Brier scores = 0.07). By machine learning, we found that the AUC of the models generated by general parameters, STE-related parameters and VFM-related parameters was higher than that of the other models, but the increase in AUC was not significant. Furthermore, analyzing and extracting data from STE and VFM techniques requires a certain amount of time and effort, which is often difficult to perform in routine clinical practice. However, with continuous attempts to explore and screen more valuable parameters, the most valuable indicators can be easily extracted from data, which is beneficial for disease assessment and clinical decision-making in patients with heart failure.

## Study limitations

There were several limitations to this study that require comment. First, this was a single-center study with a limited sample size. A larger study sample from various centers may be helpful to further confirm these observations. The findings of the present study, although showing statistical significance, were based on a relatively small number of patients and are indeed not suitable for data from countries that respond to an aging population. The results should therefore be regarded as preliminary, and further enlarge studies adequately powered for clinical outcomes are warranted to confirm our results. Second, a relatively short follow-up duration was used to detect cardiac events in this study, the effective time follow-up within 6 months is relatively short, the specific time of MACE cannot be clearly defined by patients, therefore logist regression is used to draw nomgraph this time, only to predict the possibility of MACE within 6 months after discharge. In future studies, the continuous follow-up will be summarized to establish an effective cox prediction model. Third, VFM technology relies on strict contour condition provided by the endocardial-blood interface and is limited by the frame rate applied and the Nyquist limit. Aliasing may result in errors in the VFM calculated blood flow data, mild aliasing was manually corrected on the selected frames. However, severe aliasing could not be corrected can underestimate the true velocity, we should minimize this phenomenon by optimizing the Nyquist limit and ensuring appropriate patient selection Patients with valcular heart diseases were not included in the analysis due to severe aliasing produced by the fast valve flow. Fourth, we did not include additional measurements for right ventricular function, which would need to be addressed in future investigations.

## Conclusion

In this study, we developed a prediction model and nomogram to estimate the risk of MACE within 6 months of discharge among patients with heart failure. The findings may provide a reference for clinical physicians for detection of the risk of MACE in terms of clinical characteristics, cardiac structure and function, hemodynamics, and enable its prompt management, which is a convenient, practical and effective clinical decision making tool for providing accurate prognosis.

## Data availability statement

The original contributions presented in the study are included in the article/supplementary material, further inquiries can be directed to the corresponding authors.

## Ethics statement

Written informed consent was obtained from the individual(s) for the publication of any potentially identifiable images or data included in this article.

## Author contributions

QL-S proposed the hypothesis, designed the experiments, and drafted the manuscript. QL-S, SQ-J, XD-W, and YL made contributions to data collection. QL-S and JC-Z made contributions to the analysis and interpretation of the data. JW-T and HR-L conceived the study, participated in its design, and helped edit the manuscript. All authors contributed to the article and approved the submitted version.

## Abbreviations

A: late diastolic transmitral flow velocity
a’: late diastolic relaxation velocity at septal mitral annular position
ac: atrium contraction
ANOVA: analysis of variance
ALT: alanine transaminase
ApoA: apolipoproteinA
ACEI: Angiotensin-converting enzyme inhibitors
ApoB: apolipoproteinB
AR: aortic regurgitation
ARBS: angiotensin receptor blockers
AST: aspartate transaminase
AUC: area under curve
BSA: body surface area
C2: 2-chamber
C3: 3-chamber
C4: 4-chamber
CI: cardiac index
CV: cardiovascular
CW: clockwise
CCW: counter-clockwise
DBP: diastolic blood pressure
DCM: dilated cardiomyopathy
ed: early diastole
e’: early diastolic relaxation velocity at septal mitral annular position
E: early diastolic transmitral flow velocity
EL: energy loss
ELC3P3: energy loss from 3-chamber images during isovolumic relaxation period
GDMT: Guideline Determined Medication Therapy
GHB: glycatedhemoglobin
GLS: global longitudinal strain
GLSacC2LV: left ventricular global longitudinal strain from 2-chamber images during atrial systolic period
GLSedC3LV: left ventricular global longitudinal strain from 3-chamber images during early diastolic period
GLSacLV: average of left ventricular global longitudinal strain during atrial systolic period
GLSR: global systolic strain rate
GLSRconduitC4LA: left atrial global longitudinal strain rate during conduit period
HF: heart failure
HCM: hypertrophic cardiomyopathy
HFpEF: heart failure with preserved ejection fraction
HFmrEF: heart failure with mildly reduced ejection fraction
HFrEF: heart failure with reduced ejection fraction
HFimpEF: heart failure with improved ejection fraction
HR: heart rate
HPLS: hyperlipidemia stability
ICM: ischemic cardiomyopathy
INR: international normalized ratio
IQRs: interquartile ranges
IVPD: intraventricular pressure difference
IVPDC2P4: intraventricular pressure differences from 2-chamber images during LV early filling period
IVPG: intraventricular pressure gradient
KNN: k-nearest neighbor classifier
L: lateral side
LA: left atrial
LAD: left atrium diameter
LASSO: Least Absolute Shrinkage and Selection Operator
LV: left ventricular
LVEDd: left ventricular end diastolic diameter
LVESd: left ventricular end systolic diameter
LVEF: left ventricular ejection fraction
LAVI: left atrial volume index
LVMI: left ventricular mass index
MACE: major cardiovascular events
MEL: mean energy loss
MI: myocardial infarction
MLP: Multi-LayerPerceptron
NA: not available
NT-pro BNP: N-terminal pro-brain natriuretic peptide
NYHA: New York Heart Association
P: inorganicphosphorus
P1: isovolumic contraction period
P2: ejection period
P3: isovolumetric relaxation period
P4: diastolic filling period
P5: atrial contraction period
PALS: peak atrial longitudinal strain
PALSconduitLA: Peak LA longitudinal peak strain during conduit period
s: systolic
S: septal side
SBP: systolic blood pressure
SGLT-2: sodium-dependent glucose transporters 2
STE: speckle tracking echocardiography
SVM: support vector machine
TRV: tricuspid regurgitant flow velocity
TRPD: tricuspid regurgitation pressure difference
VFM: vector flow mapping
VIF: the variance inflation factor
VorAreaC4S: LV vortex area from 4-chamber images during systolic period
XGBoost: extreme gradient boosting classifier.

## References

[1] Groenewegen A Rutten FH Mosterd A Hoes AW. Epidemiology of heart failure. *Eur J Heart Fail.* (2020) 22:1342–56. 10.1002/ejhf.1858 32483830 PMC7540043

[2] Ambrosy AP Parikh RV Sung SH Tan TC Narayanan A Masson R Analysis of worsening heart failure events in an integrated health care system. *J Am Coll Cardiol.* (2022) 80:111–22. 10.1016/j.jacc.2022.04.045 35798445 PMC10329847

[3] Lesyuk W Kriza C Kolominsky-Rabas P. Cost-of-illness studies in heart failure: a systematic review 2004-2016. *BMC Cardiovasc Disord.* (2018) 18:74. 10.1186/s12872-018-0815-3 29716540 PMC5930493

[4] Urbich M Globe G Pantiri K Heisen M Bennison C Wirtz HS A systematic review of medical costs associated with Heart Failure in the USA (2014-2020). *Pharmacoeconomics.* (2020) 38:1219–36. 10.1007/s40273-020-00952-0 32812149 PMC7546989

[5] Thenappan T Prins KW Cogswell R Shah SJ. Pulmonary hypertension secondary to heart failure with preserved ejection fraction. *Can J Cardiol.* (2015) 31:430–9. 10.1016/j.cjca.2014.12.028 25840094

[6] Francis GS Cogswell R Thenappan T. The heterogeneity of heart failure: will enhanced phenotyping be necessary for future clinical trial success? *J Am Coll Cardiol.* (2014) 64:1775–6. 10.1016/j.jacc.2014.07.978 25443697

[7] Hsu B Korda RJ Lindley RI Douglas KA Naganathan V Jorm LR. Use of health and aged care services in Australia following hospital admission for myocardial infarction, stroke or heart failure. *BMC Geriatr.* (2021) 21:538. 10.1186/s12877-021-02519-w 34635068 PMC8504055

[8] Valley TS Sjoding MW Ryan AM Iwashyna TJ Cooke CR. Intensive care unit admission and survival among older patients with chronic obstructive pulmonary disease, Heart Failure, or Myocardial Infarction. *Ann Am Thorac Soc.* (2017) 14:943–51. 10.1513/AnnalsATS.201611-847OC 28208030 PMC5566309

[9] Hsu B Korda R Naganathan V Lewis P Ooi SY Brieger D Burden of cardiovascular diseases in older adults using aged care services. *Age Ageing.* (2021) 50:1845–9. 10.1093/ageing/afab083 34146393

[10] Arnett DK Blumenthal RS Albert MA Buroker AB Goldberger ZD Hahn EJ 2019 ACC/AHA guideline on the primary prevention of cardiovascular disease: a report of the American College of Cardiology/American Heart Association task force on clinical practice guidelines. *J Am Coll Cardiol.* (2019) 74:e177–232. 30894318 10.1016/j.jacc.2019.03.010 PMC7685565

[11] Voors AA Ouwerkerk W Zannad F van Veldhuisen DJ Samani NJ Ponikowski P Development and validation of multivariable models to predict mortality and hospitalization in patients with heart failure. *Eur J Heart Fail.* (2017) 19:627–34. 10.1002/ejhf.785 28247565

[12] Lim NK Lee SE Lee HY Cho HJ Choe WS Kim H Risk prediction for 30-day heart failure-specific readmission or death after discharge: data from the Korean Acute Heart Failure (KorAHF) registry. *J Cardiol.* (2019) 73:108–13. 30360893 10.1016/j.jjcc.2018.07.009

[13] Wang L Zhao YT. Development and validation of a prediction model for irreversible worsened cardiac function in patients with acute decompensated heart failure. *Front Cardiovasc Med.* (2021) 8:785587. 10.3389/fcvm.2021.785587 34957263 PMC8702716

[14] Segar MW Jaeger BC Patel KV Nambi V Ndumele CE Correa A Development and validation of machine learning-based race-specific models to predict 10-year risk of heart failure: a multicohort analysis. *Circulation.* (2021) 143:2370–83. 10.1161/CIRCULATIONAHA.120.053134 33845593 PMC9976274

[15] Zhuang B Shen T Li D Jiang Y Li G Luo Q A model for the prediction of mortality and hospitalization in chinese heart failure patients. *Front Cardiovasc Med.* (2021) 8:761605. 10.3389/fcvm.2021.761605 34869676 PMC8639158

[16] Yin T Shi S Zhu X Cheang I Lu X Gao R A survival prediction for acute heart failure patients via web-based dynamic nomogram with internal validation: a prospective cohort study. *J Inflamm Res.* (2022) 15:1953–67. 10.2147/JIR.S348139 35342297 PMC8947803

[17] Bozkurt B Coats Andrew JS Tsutsui H Abdelhamid Ca M Adamopoulos S Albert N Universal definition and classification of heart failure: a report of the Heart Failure Society of America, Heart Failure Association of the European Society of Cardiology, Japanese Heart Failure Society and Writing Committee of the Universal Definition of Heart Failure: Endorsed by the Canadian Heart Failure Society, Heart Failure Association of India, Cardiac Society of Australia and New Zealand, and Chinese Heart Failure Association. *Eur J Heart Fail.* (2021) 23:352–80. 10.1002/ejhf.2115 33605000

[18] Lang RM Badano LP Mor-Avi V Afilalo J Armstrong A Ernande L Recommendations for cardiac chamber quantification by echocardiography in adults: an update from the American Society of Echocardiography and the European Association of Cardiovascular Imaging. *J Am Soc Echocardiogr.* (2015) 28:1–39.e14. 10.1016/j.echo.2014.10.003 25559473

[19] Nagueh SF Smiseth OA Appleton CP Byrd BF Dokainish H Edvardsen T Recommendations for the evaluation of left ventricular diastolic function by echocardiography: an update from the american society of echocardiography and the european association of cardiovascular imaging. *Eur Heart J Cardiovasc Imaging.* (2016) 17:1321–60. 10.1093/ehjci/jew082 27422899

[20] Unger ED Dubin RF Deo R Daruwalla V Friedman JL Medina C Association of chronic kidney disease with abnormal cardiac mechanics and adverse outcomes in patients with heart failure and preserved ejection fraction. *Eur J Heart Fail*. (2016) 18:103–12. 10.1002/ejhf.445 26635076 PMC4713321

[21] Mirea O Duchenne J Voigt JU. Comparison between nondedicated and novel dedicated tracking tool for right ventricular and left atrial strain. *J Am Soc Echocardiogr.* (2022) 35:419–25. 10.1016/j.echo.2021.11.011 34800672

[22] Asami R Tanaka T Shimizu M Seki Y Nishiyama T Sakashita H Ultrasonic vascular vector flow mapping for 2-D flow estimation. *Ultrasound Med Biol.* (2019) 45:1663–74. 10.1016/j.ultrasmedbio.2019.02.014 31003710

[23] Garcia D Del Alamo JC Tanne D Yotti R Cortina C Bertrand E Two-dimensional intraventricular flow mapping by digital processing conventional color-Doppler echocardiography images. *IEEE Trans Med Imaging.* (2010) 29:1701–13. 10.1109/TMI.2010.2049656 20562044

[24] Stugaard M Koriyama H Katsuki K Masuda K Asanuma T Takeda Y Energy loss in the left ventricle obtained by vector flow mapping as a new quantitative measure of severity of aortic regurgitation: a combined experimental and clinical study. *Eur Heart J Cardiovasc Imaging.* (2015) 16:723–30. 10.1093/ehjci/jev035 25762562

[25] Asami R Tanaka T Kawabata KI Hashiba K Okada T Nishiyama T. Accuracy and limitations of vector flow mapping: left ventricular phantom validation using stereo particle image velocimetory. *J Echocardiogr.* (2017) 15:57–66. 10.1007/s12574-016-0321-5 27848215 PMC5429903

[26] Du Y Goddi A Bortolotto C Shen Y Dell’Era A Calliada F Wall shear stress measurements based on ultrasound vector flow imaging: theoretical studies and clinical examples. *J Ultrasound Med.* (2020) 39:1649–64. 10.1002/jum.15253 32124997 PMC7497026

[27] Rodríguez Muñoz D Moya Mur JL Fernández-Golfín C Becker Filho DC González Gómez A Fernández Santos S Left ventricular vortices as observed by vector flow mapping: main determinants and their relation to left ventricular filling. *Echocardiography.* (2015) 32:96–105. 10.1111/echo.12584 24661050

[28] Ji L Hu W Yong Y Wu H Zhou L Xu D. Left ventricular energy loss and wall shear stress assessed by vector flow mapping in patients with hypertrophic cardiomyopathy. *Int J Cardiovasc Imaging.* (2018) 34:1383–91. 10.1007/s10554-018-1348-7 29626283

[29] Chen M Jin JM Zhang Y Gao Y Liu SL. Assessment of left ventricular diastolic dysfunction based on the intraventricular velocity difference by vector flow mapping. *J Ultrasound Med.* (2013) 32:2063–71. 10.7863/ultra.32.12.2063 24277887

[30] Harrell FE Lee KL Mark DB. Multivariable prognostic models: issues in developing models, evaluating assumptions and adequacy, and measuring and reducing errors. *Stat Med.* (1996) 15:361–87. 10.1002/(SICI)1097-0258(19960229)15:4<361::AID-SIM168>3.0.CO;2-4 8668867

[31] Malagoli A Rossi L Bursi F Zanni A Sticozzi C Piepoli MF Left atrial function predicts cardiovascular events in patients with chronic heart failure with reduced ejection fraction. *J Am Soc Echocardiogr.* (2019) 32:248–56. 10.1016/j.echo.2018.08.012 30316541

[32] Collins FS Varmus H. A new initiative on precision medicine. *N Engl J Med.* (2015) 372:793–5. 10.1056/NEJMp1500523 25635347 PMC5101938

[33] Matsuda Y Toma Y Ogawa H Matsuzaki M Katayama K Fujii T Importance of left atrial function in patients with myocardial infarction. *Circulation.* (1983) 67:566–71. 10.1161/01.cir.67.3.566 6821898

[34] Rosca M Lancellotti P Popescu BA Piérard LA. Left atrial function: pathophysiology, echocardiographic assessment, and clinical applications. *Heart.* (2011) 97:1982–9. 10.1136/heartjnl-2011-300069 22058287

[35] Chrysohoou C Kotroyiannis I Antoniou CC Brili S Vaina S Latsios G Left atrial function predicts heart failure events in patients with newly diagnosed left ventricular systolic heart failure during short-term follow-up. *Angiology.* (2014) 65:817–23. 10.1177/0003319713506109 24165115

[36] Sanchis L Andrea R Falces C Lopez-Sobrino T Montserrat S Perez-Villa F Prognostic value of left atrial strain in outpatients with de novo heart failure. *J Am Soc Echocardiogr.* (2016) 29:1035–42.e1. 10.1016/j.echo.2016.07.012 27624593

[37] Pellicori P Zhang J Lukaschuk E Joseph AC Bourantas CV Loh H Left atrial function measured by cardiac magnetic resonance imaging in patients with heart failure: clinical associations and prognostic value. *Eur Heart J.* (2015) 36:733–42. 10.1093/eurheartj/ehu405 25336215

[38] Tsang TS Barnes ME Gersh BJ Bailey KR Seward JB. Left atrial volume as a morphophysiologic expression of left ventricular diastolic dysfunction and relation to cardiovascular risk burden. *Am J Cardiol.* (2002) 90:1284–9. 10.1016/s0002-9149(02)02864-3 12480035

[39] Hung MJ Cherng WJ. Analysis of left atrial volume change rate for evaluation of left ventricular diastolic function. *Echocardiography.* (2004) 21:593–601. 10.1111/j.0742-2822.2004.03154.x 15488086

[40] Mottram PM Marwick TH. Assessment of diastolic function: what the general cardiologist needs to know. *Heart.* (2005) 91:681–95. 10.1136/hrt.2003.029413 15831663 PMC1768877

[41] Cui X Thunström E Dahlström U Zhou J Ge J Fu M. Trends in cause-specific readmissions in heart failure with preserved vs. reduced and mid-range ejection fraction. *ESC Heart Fail.* (2020) 7:2894–903. 10.1002/ehf2.12899 32729678 PMC7524131

[42] Rodriguez Muñoz D Markl M Moya Mur JL Barker A Fernández-Golfín C Lancellotti P Intracardiac flow visualization: current status and future directions. *Eur Heart J Cardiovasc Imaging.* (2013) 14:1029–38. 10.1093/ehjci/jet086 23907342 PMC3806582

[43] Uejima T Koike A Sawada H Aizawa T Ohtsuki S Tanaka M A new echocardiographic method for identifying vortex flow in the left ventricle: numerical validation. *Ultrasound Med Biol.* (2010) 36:772–88. 10.1016/j.ultrasmedbio.2010.02.017 20381947

[44] Li C Zhang J Li X Zhou C Li H Tang H Quantification of chronic aortic regurgitation by vector flow mapping: a novel echocardiographic method. *Eur J Echocardiogr.* (2010) 11:119–24. 10.1093/ejechocard/jep175 19933519

[45] Kainuma A Itatani K Akiyama K Naito Y Ishii M Shimizu M Preoperative left ventricular energy loss in the operating theater reflects subjective symptoms in chronic aortic regurgitation. *Front Surg.* (2022) 9:739743. 10.3389/fsurg.2022.739743 35252323 PMC8889468

[46] Cho JS Shrestha S Kagiyama N Hu L Ghaffar YA Casaclang-Verzosa G A network-based “Phenomics” approach for discovering patient subtypes from high-throughput cardiac imaging data. *JACC Cardiovasc Imaging.* (2020) 13:1655–70. 10.1016/j.jcmg.2020.02.008 32762883

